# A scoping review of female drowning: an underexplored issue in five high-income countries

**DOI:** 10.1186/s12889-021-10920-8

**Published:** 2021-06-05

**Authors:** Kym Roberts, Ogilvie Thom, Susan Devine, Peter A. Leggat, Amy E. Peden, Richard C. Franklin

**Affiliations:** 1 Emergency Department, Sunshine Coast University Hospital, Sunshine Coast Hospital and Health Service, Sunshine Coast, Queensland Australia; 2 College of Public Health, Medical and Veterinary Sciences, James Cook University, Townsville, Queensland Australia; 3 School of Medicine, National University of Ireland Galway, Galway, Ireland; 4 School of Population Health, University of New South Wales, Sydney, New South Wales Australia

**Keywords:** Drowning, Female, Adult, Clinical treatment, Clinical outcomes, Risk factors, Prevention

## Abstract

**Background:**

Drowning is a significant public health issue, with females accounting for one third of global drowning deaths. The rate of female drowning has not decreased within high-income countries and presentations to hospital have increased. This scoping review aimed to explore adult female unintentional drowning, including risk factors, clinical treatment and outcomes of females hospitalised for drowning.

**Methods:**

A systematic search of the literature following the PRISMA-ScR framework was undertaken. The databases OVID MEDLINE, Embase, CINAHL, OVID Emcare, Web of Science, Informit and Scopus were accessed. Study locations of focus were Australia, Canada, New Zealand, the United Kingdom, and the United States. Studies from January 2003 to April 2019 were included. The quality of evidence of included studies was assessed using GRADE guidelines.

**Results:**

The final search results included 14 studies from Australia (*n* = 4), Canada (*n* = 1), New Zealand (n = 1), United States (*n* = 6), United Kingdom (n = 1), and one study reporting data from both Australia and United States. Nine studies reported risk factors for female drowning including age, with the proportion of female drowning incidence increasing with age. Although females are now engaging in risk-taking behaviours associated with drowning that are similar to males, such as consuming alcohol and swimming in unsafe locations, their exposure to risky situations and ways they assess risk, differ. Females are more likely to drown from accidental entry into water, such as in a vehicle during a flood or fall into water. This review found no evidence on the clinical treatment provided to females in hospital after a drowning incident, and only a small number of studies reported the clinical outcomes of females, with inconsistent results (some studies reported better and some no difference in clinical outcomes among females).

**Conclusion:**

Adult females are a group vulnerable to drowning, that have lacked attention. There was no single study found which focused solely on female drowning. There is a need for further research to explore female risk factors, the clinical treatment and outcomes of females hospitalised for drowning. This will not only save the lives of females, but also contribute to an overall reduction in drowning.

**Supplementary Information:**

The online version contains supplementary material available at 10.1186/s12889-021-10920-8.

## Background

Drowning is a significant public health issue. Globally, drowning is the third leading cause of injury related death [1, 2]. In 2017, there were an estimated 295,000 unintentional drowning deaths globally, with males and children experiencing the highest rates [1–3]. Although the proportion of drowning among females is one third of global drowning deaths [3], the literature on drowning is inequitably predominated by a focus on children and males [4].

The female rate of drowning does not appear to be decreasing, and evidence shows recent increases in the number of female drowning fatalities within high-income countries (HIC) [5–7]. Both Australia and New Zealand have experienced a 2 % increase in the number of female drowning fatalities in 2018 compared with 2017 [6, 8]. Meanwhile, across the same periods, the number of overall drowning fatalities decreased by 14% in Australia [9] and 27% in New Zealand [6].

The proportion of females hospitalised due to a drowning incident has increased in recent years in Australia, New Zealand, and the United States [5–7]. Females accounted for 34% of non-fatal drowning incidents in Australia from 2002 to 2015, peaking at 38% in 2010 to 2011 [5]. For every five female patients admitted to hospital for drowning in Australia, there is one fatality and four survivors [5]. This ratio of fatalities to survivors for hospital drowning admissions is almost half that of males, at one fatality for every 2.4 survivors [5].

There are gender and sex differences in the risk factors and causes of drowning, with males known to engage in higher risk-taking behaviours compared to females [10, 11]. Males have increased participation in aquatic activities, are more confident in their swimming skills, are more likely to swim alone, and more likely to use alcohol in the aquatic environment compared to females [10]. However, these factors alone do not fully explain the differences in the fatal and non-fatal drowning rates between males and females, although exposure is likely to play a role [12, 13]. An Australian study identified that females were more likely to drown as a result of unintentional exposure to rivers, such as being swept away by floodwaters, when compared to males [14]. Drowning risk for males is associated with intentional activities involving water exposure such as fishing, jumping into water and using watercraft [10].

Studies in the area of emergency medicine and critical care have found being female is a protective factor in the outcome of cardiac arrest, trauma and post-traumatic sequelae, such as pneumonia, sepsis and organ failure [15–19]. This also appears to be similar in drowning literature with females experiencing improved outcomes compared to males, when hospitalised for drowning [5, 20, 21]. Clinical studies in emergency and critical care medicine have suggested the improvement in outcomes among females may be due to the role of female sex hormones with oestrogen producing cellular changes and influencing the inflammatory cascade in response to injury and critical illness [15]. Female sex hormones may also be protective in the outcomes of global hypoxia and may decrease secondary cardiovascular and neurological ischaemia [22]. Knowing more about female drowning risk factors and treatment will help to inform prevention and may also help to improve outcomes for other high-risk groups such as males. Emergency medicine and gender and sex health researchers have advocated for an increased focus and reporting in clinical studies on gender and sex based differences, believing this will benefit the health outcomes of both sexes [23, 24].

The impact of unintentional injury among females is a research area that is still largely unexplored [25]. This scoping review aimed to explore the evidence on female unintentional drowning, including risk factors and the clinical treatment and outcomes of females after presentation to the emergency department (ED) and hospital admission.

## Methods

A review of the literature using a systematic approach following the Preferred Reporting Items for Systematic Reviews and Meta-Analysis extension for Scoping Reviews (PRISMA-ScR) framework [26] was undertaken to identify, explore and situate the research data published on female drowning focusing on the risk factors, clinical treatment and outcomes. The framework by The Johanna Briggs Institute ‘Methodology for JBI Scoping Reviews’ and the five step process outlined by Arskey and O’Malley (2005) was also followed [27, 28].

This review was designed to address the following research questions:

1. What is the evidence that adds to our understanding of risk factors for unintentional female drowning?
2. What evidence has been reported from primary studies on the clinical treatment and outcomes of females presenting to the ED and hospitalised for drowning?

Study selection criteria was developed for the literature search by defining the inclusion and exclusion criteria (Table 1). The definition of non-fatal drowning in this review was patients treated in the ED and hospitalised for an unintentional drowning-related incident.

**Table 1:** Study inclusion and exclusion criteria

Inclusion	Exclusion
Clinical treatment and outcomes of female adults treated in the emergency department (ED) and hospitalised for drowning	Studies where adults and children could not be separated in the data
Epidemiology (age and proportion by gender and sex) and risk factors for unintentional female fatal and non-fatal drowning (were included if they added value to our understanding)	Case reports, government reports, conference abstracts, literature reviews, editorials, policy statements, letters
Adults (18 years or older)	Children (less than 18 years of age)
Study locations: Australia, New Zealand, United States, United Kingdom and Canada	Countries outside the chosen study locations
Published in English in peer-reviewed journal	Published in a language other than English and/or not peer-reviewed
Date: January 2003 to April 2019	Date: Studies published before January 2003

Studies were included only where they added value to our understanding of risk factors. For example, if a paper only had information about the male to female ratio, then this was not included; however, if it had a breakdown by gender and sex and activity then this was included. Studies reporting the epidemiology of drowning, for example, age and proportion of drowning incidents by gender and sex were included in the review where they added value to our understanding of risk factors.

Five high income countries (HICs), namely Australia, Canada, New Zealand, the United Kingdom, and the United States, were chosen as study locations of interest for this review due to a similar standard of medical care and similar cultural engagement in aquatic activities [29, 30]. Studies published from January 2003 to April 2019 were included in the review. This time period was chosen to explore the current medical treatment for drowning reflected by the ‘Recommended Guidelines for Uniform Reporting of Data from Drowning’ issued in 2003 [31], the 2005 statement on the revised drowning definition [32], and changes made to cardiopulmonary resuscitation guidelines in 2005 by the International Liaison Committee on Resuscitation (ILCOR) [20, 33].

A search strategy was developed to identify relevant primary studies, with the first step to conduct an electronic database search, followed by a manual search of reference lists of included publications to identify additional relevant publications (see Additional File 1). The databases OVID MEDLINE, Embase, CINAHL, OVID Emcare, Web of Science, Informit and Scopus were accessed, systematic search of the databases was conducted from 29 April to 22 May 2019. The focus of the search strategy was to identify studies on unintentional fatal and non-fatal drowning focused on females, or studies that added value to the evidence on females involved in drowning incidents, or studies on unintentional drowning that included sex as a variable in the results. When included studies reported only on males and did not present results for females, we would calculate the inferred results for females.

**Fig. 1:**
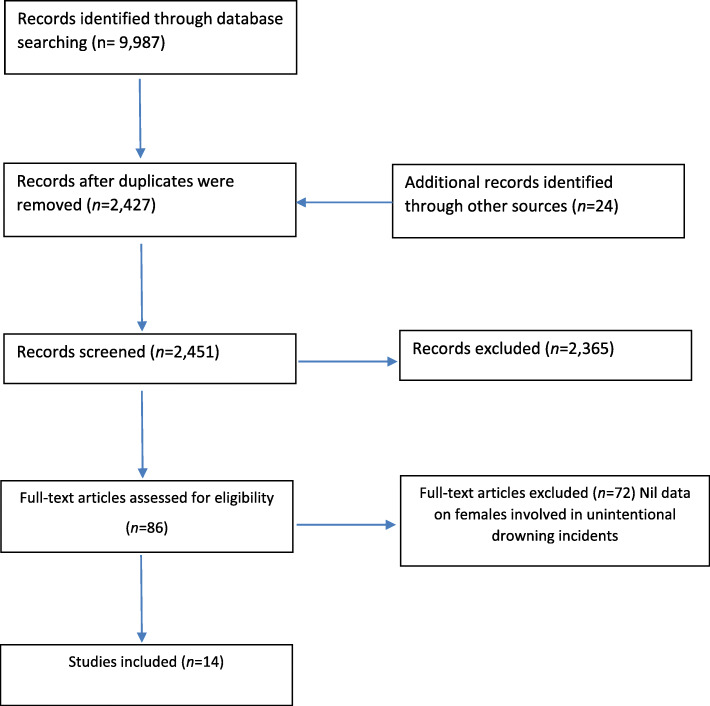
Modified PRISMA flowchart

Two reviewers (first and second author) applied the inclusion and exclusion criteria to assess for eligibility of the primary studies. Any disagreements were referred to a third author for resolution, who also spot checked the excluded references. Duplicates within the search library were identified and excluded and a review of search results was conducted first by title, abstract and then full-text article review (Fig. 1).

The Australian National Health and Medical Research Council’s (NHMRC) Evidence Hierarchy [34] and the Grading of Recommendations Assessment, Development and Evaluation (GRADE) guidelines [35] were utilised to identify the level and quality of evidence of the study designs of the included papers. The NHMRC evidence hierarchy defines four levels of evidence from I (high quality of evidence) to IV (low level of evidence) based on the fit of the research question and study design to appropriately answer the research question and minimize study bias [34]. The GRADE guidelines recommend observational studies are rated as having low quality of evidence, and then assessed according to study limitations (risk of bias), inconsistency, indirectness, imprecision and publication bias [35]. The GRADE evaluation profile for the included studies can be found in Supplementary File 1 (Additional file 2).

Data extracted from the included studies (author, study location, study aim, study design/methodology, study sample, results, and limitations) are summarized in Tables 2 and 3. The results of the database searches were collated, summarized and reported in a descriptive format. This provided a framework to identify the main research themes [40] and identify the gaps and limitations in the existing evidence on female drowning.

**Table 2:** Included papers on unintentional female drowning epidemiology and risk factors (*n*=9) (Results, page 10)

Author	Country	Study aim	Study design/methodology	Study sample	Results	Risk factor	Evidence hierarchy (34)/GRADE [35]	Limitations
Peden et al. (2016) [14]	Australia	Unintentional drowning deaths in rivers, creeks and streams	*Design:* Cross-sectional study *Data collection:* Australian National Coronial Information System, July 2002 to June 2012.	Drowning deaths: Females *n*=151/770 (19.6%)	Females 2.27 times more likely to drown in rivers in non-aquatic transport, and 4.45 times more likely to drown being swept away by floodwaters.	Activity: non-aquatic transport, being swept away	Level IV-Low level evidence GRADE-Very low	Coroner cases with open findings may overestimate number of deaths from river drownings. Some variables had limited information due to open finding.
Gulliver & Begg (2005) [41]	New Zealand	To describe water-related behaviour and non-fatal drowning incidents among young adults	*Design:* Cross-sectional study *Data collection:* Longitudinal study, Dunedin Multidisciplinary Health and Development Study, 1993 to 1994.	Non-fatal drowning incidents: Females *n*=52/141 (37%)	Females reported lower rates of water confidence, risk exposure and experience of a non-fatal drowning incident. Females were higher risk of drowning within two hours of consuming alcohol and engaging in watercraft activity.	Water confidence, exposure, alcohol and watercraft	Level IV-Low level evidence GRADE-Very low	Participants were asked about alcohol use and engagement in water activity, although the amount of alcohol consumption was not recorded.
Henderson & Wilson (2006) [42]	United Kingdom	To examine hospital admissions from a water-related incident.	*Design:* Cross-sectional study *Data collection*: HES England using ICD-10 coding, 1997 to 2004.	Females *n*=1787/6793 (26.3%)	1 fatal drowning=3 hospital presentations. Numbers of females increased in the ICD-10 codes for: drowning and submersion in bathtub and following fall into bathtub, and victim of flood.	Submersion in bathtub, fall into bathtub and victim of flood	Level IV-Low level evidence GRADE-Very low	Personal and environmental factors prior to drowning incident were not captured in this study.
Hudson et al. (2006) [43]	United States	To examine factors associated with injuries occurring in drowning incidents among hospitalised patients in Alaska	*Design:* Cross-sectional study *Data collection*: State of Alaska Department of Public Health Alaska Trauma Registry, 1991 to 2000.	Immersion only: females *n*=25/89 (28%) Associated injuries: Females *n*=17/87 (20%)	Females associated with increased hospitalisations from a drowning incident (p=0.02). Females were found to be at higher risk of an associated injury than males.	High risk of injury from drowning	Level IV-Low level evidence GRADE-Very low	The higher risk of associated injury from immersion-related event among females may be due to Alaskan males being more experienced in working and recreationally interacting with the water.
Morgan et al. (2009) [44]	Australia	To explore self-reported water exposure, activity, protective factors and drowning risk at surf beaches by gender.	*Design:* Prospective cross-sectional study *Data collection:* Self-reported survey from December 2003 to February 2004	Females *n*=210/406 (51.7%)	Females visited surf beaches less than males, but length of stay was similar. Number of males and females engaged in wave swimming were similar (females *n*=80, males *n*=88).	Exposure, risk-taking behaviour	Level IV-Low level evidence GRADE-Very low	Sampling method may have been biased, as a person may have visited the surf beach more than once during data collection (16 days). Small sample size in sub-group for surfing (females *n*=14, males *n*=79).
Nasrullah & Muazzam (2011) [45]	United States	To describe demographics and changes in unintentional drowning mortality	*Design:* Cross-sectional study *Data collection*: Centers for Disease Control and Prevention Web-based Injury Statistics Query and Reporting System mortality data. 1999 to 2006	Females *n*=5846/27514 (21.2%)	Increase of 9.5% of drowning fatalities for females, male drowning fatalities decreased by 0.7%. Female drowning fatalities increased by 7% in 2004 to 2005	Fatal drowning	Level IV-Low level evidence GRADE-Very low	There may be errors or missing data on death certificates that could lead to an incorrect classification of injury.
Peden et al. (2018) [46]	Australia	To explore river use and alcohol consumption and attitudes towards drowning risk	*Design:* Prospective Cross-sectional study *Data Collection:* Convenience sample, survey conducted in four river locations across Australia	Females *n*=353/684 (51.6%)	Females visiting rivers in similar number to males but females engaging in non-aquatic activities. Higher number of females with positive blood concentration than males	Exposure, alcohol, risk-taking behaviour	Level IV-Low level evidence GRADE-Vey low	Possibility of recall bias due to self-reporting survey. Random convenience sampling used, and refusal rate was not documented.
Peden et al. (2018) [47]	Australia	To compare fatal and non-fatal drowning databases in Australia to identify key ratios, differences and inform drowning prevention strategies.	*Design:* Retrospective Cross-sectional study *Data collection:* Royal Life Saving Society Australia National Fatal Drowning Database and Australian Institute of Health and Welfare National Hospital Morbidity Database, July 2002 to June 2015.	Fatalities (all ages): Females *n*=525/2272 (23.1%) Hospital separations (all ages): Females *n*=2088/6158 (33.9%)	Unintentional fatal drowning: Adult females *n*=353/1737 (20.3%) Hospitalisations for drowning: Adult females *n*=687/2585 (26.6%)	Non-fatal drowning	Level IV-Low level evidence GRADE-Very low	An error could have occurred during in the prediction of fatal drownings, a correction factor was applied to the number of fatal drowning incidents and to predict the number of non-fatal drowning incidents. ICD coding for drowning data was limited to the codes W65-74 as primary cause of hospitalisation for non-fatal drowning incidents.
Clemens et al. (2016) [48]	Canada	To describe the characteristics of drowning fatalities by age	*Design:* Retrospective descriptive analysis *Data collection:* Coroner’s reports, hospital data, police reports and death certificates, January 2008 to December 2012.	Females *n*=424/2391 (17.7%)	Drowning incidents: adult females: 20-34 years: *n*=73/592 (12.3%), 35-64 years: *n*=179/1033 (17.3%), 65 + years: *n*=93/419 (22.2%) Female adult unintentional drowning deaths*: 20-34 years: (0.42), 35-64 years: (0.5), 65 + years: (0.69)	Age	Level IV-Low level evidence GRADE-Very low	Risk of information bias due to data recorded by individual data collectors. Some data missing

**Table 3:** Included papers on female drowning clinical treatment and outcomes (*n* = 5)

Author	Country	Study aim	Study Design/Methodology	Study sample	Results	Evidence Hierarchy [34]/GRADE [35]	Limitations
El Sibai et al. (2018) [4]	United States	To describe the characteristics and predictors of poor outcome among ED presentations for drowning	*Design:* Retrospective cross-sectional study *Data Collection*: NEDS dataset, 2013	Females *n* = 4283/12529 (34.2%)	Multivariate analysis results report significant positive predictor of poor outcomes among males. Over half of patients were discharged home from ED.	Level IV-Low level evidence GRADE-Very low	NEDS data represents 20% of US hospital based ED’s. Errors reported in injury coding for submersion therefore some drownings missed.
Lee et al. (2006) [36]	United States	To describe the epidemiology and outcomes of serious paediatric submersion, and identify factors associated with increased mortality and morbidity.	*Design:* Retrospective case-series *Data collection:* Massachusetts electronic death database from Department of Public Health Registry of Vital Records and Statistics, and Massachusetts Hospital discharge Database from 1994 to 2000. Ages 0–19 years	Females *n* = 89/267 (33%)	Males were 2.52 times more likely to have a poor outcome than females (mortality and morbidity)	Level IV-Low level evidence GRADE-Very low	Limitations in data collected from databases such as quality, duplicates or missed cases. Data only contained patients admitted to hospital wards as ED state-wide data was not available.
Quan et al. (2014) [37]	United States, United Kingdom, Australia	To assess the association between water temperature and duration of submersion in the outcome of drowning.	*Design:* Case-control study *Data collection:* Western Washington database, January 1974 to June 1996. Data collection was limited to unintentional drowning that occurred in open waters (lakes, rivers and oceans)	Females *n* = 161/1094 (15%)	Good outcome (survived with or without limited neurological deficit or injury): Females *n* = 69/276 (24.9%) Good outcome associated with age less than 15 years, female and immersion duration of less than 6 min in water greater than 16 degrees Celsius.	Level III-3-Low level evidence GRADE-Very low	Retrospective data collection limitations with hand searching, although authors believe high case ascertainment from this method. Water temperatures were estimated, as not routinely collected at the time of the drowning incident.
Reynolds et al. (2017) [38]	United States	To estimate long-term mortality and identify prognostic factors in drowning victims.	*Design:* Cohort study *Data collection:* Western Washing Drowning Registry on non-fatal drowning incidents from January 1974 to July 1996.	Females *n* = 247/776 (31.8%)	Long-term mortality: univariate analysis for Male sex –non-significant (Cox proportional hazard modelling).	Level III-3-Low level evidence GRADE-Very low	Difficult to predict long-term survival from drowning event due to accuracy of coding data, although this association suggested.
Reynolds et al. (2019) [39]	United States	To estimate long-term survival after cardiac arrest from drowning.	*Design:* Cohort Study *Data collection:* Western Washington Drowning Registry on non-fatal drowning incidents. January 1974 to July 1996.	Females *n =* 109/407 (26.8%) (Survived to hospital admission)	Long-term survival: Females *n* = 18/54 (33.3%)	Level III-3-Low level evidence GRADE-Very low	Missing data, only variables with less than 25% missing data were included in multivariate analysis.

Legend: *ED* emergency department; *GRADE* Grading of Recommendations Assessment, Development and Evaluation guidelines; *NEDS* Nationwide Emergency Department Sample (USA)

## Results

The systematic search of the databases revealed 9987 papers, after duplicates were removed, a review of the papers by title and then abstract was conducted, this left 86 papers for full text review (Fig. 1). Seventy-two papers were excluded after full-text review, a total of 14 articles met the inclusion criteria for the scoping review [4, 14, 36–39, 41–48]. The final search results included studies from Australia (*n* = 4) [14, 44, 46, 47], Canada (*n* = 1) [48], New Zealand (n = 1) [41], the United States (*n* = 6) [4, 36, 38, 39, 43, 45], and the United Kingdom (n = 1) [42], with one study reporting data from both Australia and the United States [37]. The study designs of the included papers, according to NHMRC evidence hierarchy [34], were all low evidence studies. This included level III-3 evidence cohort studies (*n* = 2) [38, 39] and case-control study (n = 1) [37], level IV evidence case-series (n = 1) [36], cross-sectional studies (*n* = 9) [4, 14, 42–47] and a descriptive study (n = 1) [48].

### Unintentional female drowning epidemiology and risk factors

Nine studies [14, 41–48] reported on the epidemiology (age and proportion of incidence by gender and sex) and risk factors for adult female unintentional drowning Tables 2 and 3. The proportion of female drowning fatalities was similar in Australia, Canada and United States [45, 47, 48]. Females accounted for 20% of drowning deaths in Australia from 2002 to 2015 [47], 18% for Canada from 2008 to 2012 [48], and 21% for USA from 1999 to 2006 [45]. In the United States female drowning fatalities increased by 10% during this period and fatalities among males decreased by 1% [45]. The proportion of drowning fatalities increased with age among females in Canada during 2008 to 2012, from 12% (73/592) in those aged 20–34 years, to 17% (179/1033) in those aged 35–64 years, and peaking at 22% (93/419) in those aged 65 years and over [48].

Four studies [41–43, 47] reported the proportion of non-fatal drowning incidents among females from Australia, United States, United Kingdom and New Zealand. In Australia, females accounted for 27% of all hospitalisations for drowning between 2002 and 2015 [47]. An Alaskan study reported 28% of all hospital presentations for drowning were female from 1991 to 2000, but females were more likely than males to be admitted to hospital after a drowning incident (*p* = 0.02) [43]. In the United Kingdom during 1997 to 2004, 26% of hospital admissions for drowning were female [42]. A study from New Zealand found that 37% of females reported they had been involved in a drowning incident [41].

Three studies [41, 44, 46] reported risk-taking behaviours, protective factors and risk exposure among females. Females were found to be visiting rivers in similar proportions to males [46]. One study found the amount of time visiting the beach was similar for females and males, but females more commonly participated in low-risk aquatic activities such as wading and staying close to the shore [44]. Females reported they were less confident engaging in aquatic activities compared to males [41, 44] and returning to shore, if caught in a rip [44]. Females did not differ from males in self-protective behaviours to avoid drowning incidents such as using a flotation device and undertaking first aid training [41, 44]. Females, who felt confident with their swimming skills, engaged in similar risk-taking behaviours to males, such as swimming in unsafe aquatic locations [41]. An Australian study found the number of females visiting rivers and the level of alcohol ingestion among females was similar to males, increasing the risk of drowning [46]. Interestingly, females in the study underestimated their level of alcohol ingestion, when compared to blood alcohol concentration [46]. A study from New Zealand found females experienced a higher risk of drowning, such as from falling from watercraft, within two hours of ingesting alcohol and engaging in a watercraft activity [41].

Two studies found females were more likely to drown from accidental, rather than intentional, entry into water which differed from males [14, 42]. A study of patients hospitalised for drowning from the United Kingdom found females more commonly experienced a drowning incident while in a bathtub, from a fall into bathtub, and being the victim of a flood [42]. An Australian study on drowning in rivers found females were twice as likely to be involved in a drowning incident involving non-aquatic transport, such as motor vehicles, and four times more likely due to accidental entry into flood waters [14].

### Clinical treatment and outcomes

Five studies [4, 36–39] reported the clinical outcomes of females hospitalised due to non-fatal drowning incidents, while none of the studies reported on the clinical treatment provided to females after drowning (Table 3). All studies reported data from the United States. One ED based study found females had a better outcome for morbidity and death after treatment in the ED for drowning [4]. All patients had an average length of stay in hospital of 4 days, with 60% of patients treated and discharged from ED without requiring inpatient admission [4]. Lee et al. [36] found females were more likely to experience a better outcome than males for morbidity and mortality after treatment in hospital for drowning. A study by Quan et al. [37] reported on drowning outcomes of hospitalised patients between 1975 and 1996. They found females experienced an improved neurological outcome compared to males [37]. In this study females who survived were more likely to experience no symptoms or mild symptoms of neurological injury after a drowning incident compared to males [37]. Two studies [38, 39] observed long-term mortality in patients who had survived a drowning incident. The prediction of long-term survival by sex was not significant at *p* = 0.33 [39].

## Discussion

Females are the forgotten risk group in unintentional drowning, with limited reporting on female drowning risk factors and clinical treatment and outcomes. There were no papers found from the five HICs (Australia, Canada, New Zealand, the United Kingdom, and the United States) with a singular focus on females involved in drowning incidents. The articles included in this review found the drowning risk factors for females are different to males in exposure to risk [14, 42, 44] and confidence in swimming and water skills [41]. However, in terms of risk-taking behaviours that are associated with drowning, females are increasingly engaging in similar patterns of behaviour to males, such as consuming alcohol prior to aquatic activity [41, 44, 46]. This review found no evidence from the five HICs on the clinical treatment provided to females in hospital after a drowning incident, and only a small number of studies reported the clinical outcomes of females hospitalised for drowning [4, 36–39]. However, other countries including France, Sweden, Korea and Israel, have produced studies that report on the clinical treatment of females [49, 50] and the clinical outcomes of females after drowning, such as from cardiac arrest from drowning [20, 51, 52], pneumonia [53, 54] and acute kidney injury (AKI) [21]. However, findings from these studies are limited.

### Risk factors

Adult females have recently experienced an increase in the number of unintentional non-fatal and fatal drowning incidents within the study locations examined in this review, while in the same period there was a decrease in drowning incidents among males [7, 45]. Reasons for this are not evident. There seems to be a difference in the proportion of female drowning deaths around the world with females accounting for 33% of worldwide drowning deaths, 24% of drowning deaths in HICs, and 34% in low to middle income countries (LMIC) [3]. This proportion of female drowning is significantly higher in LMICs compared to HICs [3], and signifies the need for drowning research to focus on female drowning worldwide and not just HICs.

While this paper explored the impact of drowning on females in five HIC countries, we note that the circumstances leading to fatal and non-fatal drowning among females within these locations may not be reflective in other cultures and countries, including those from LMICs [55–57]. Drowning risk factors for females in LMICs (including Bangladesh, India, and the Philippines) may be more likely to be due to residing in close proximity to natural water bodies (with a lack of barriers or covering) and undertaking activities related to daily living, and also due to water-related transport and occupational risks [55–57]. Whereas for females residing in HICs the circumstances leading to a drowning incident are more likely due to participation in recreational aquatic activities [55].

The proportion of female drowning fatalities have also been found to increase with age, particularly among older adult females aged 65 years and over [48]. Whether this is due to females living longer than males, or risk factors particular to older females, has yet to be thoroughly explored [58]. Together these findings identify females as a vulnerable population group in drowning and emphasizes the need for drowning prevention strategies to target females [46, 59]; however, this is currently not addressed in drowning prevention campaigns.

Females may be more cautious in interpreting and assessing risk factors associated with drowning compared to males [13]. This is demonstrated by an Australian study that found females spent a similar amount of time in the water as males but more commonly engaged in low-risk activities at surf beaches, such as wading and standing in shallow water [44]. In exposure to risk, females also visit beaches and rivers in similar proportions to males [12, 46, 59]. Although the female drowning rate is lower than males, risk and exposure alone do not explain this difference, as behaviour is also an important factor in drowning risk [13]. Females are also engaging in high-risk behaviours and activities in aquatic locations similar to males, such as ingesting alcohol in aquatic locations [46], engaging in wave swimming [44], and swimming in unsafe aquatic locations [41]. The use of alcohol in one study was associated with drowning risk among females, who drink and accidently fall from watercraft [41].

The precipitating event prior to an unintentional drowning incident among females is different to males [14, 42]. Adult females were more likely to die or be admitted to hospital by accidently entering water in non-aquatic transport, such as in a motor vehicle, being the victim of a flood, or falling into water [14, 42]. For males, drowning risk is associated with activities involving water exposure such as fishing and boating [14, 42]. Further exploration of the factors involving pre-event activities that females engage in that exposes them to an increased risk of drowning is required. These results signify the need for further research exploring the risk factors for drowning among females including exposure and risk-taking behaviours and activities that females engage in at aquatic locations. Adult females should be included as a target group in drowning prevention campaigns [59] on the risks specific to them, such as being swept away in motor vehicles during floods, falling into water, and also targeting alcohol use [46].

### Clinical treatment and outcomes

The clinical management of patients presenting to the ED after a drowning incident encompasses treating the effects of global hypoxia [60]. The evidence for the clinical treatment for drowning, including resuscitation of the victim, has primarily been sourced from low-level evidence studies, mainly case series, and from studies on children [60]. Therefore, this is a limitation already existing within drowning literature and evidence on clinical treatment has primarily come from data on the therapeutic management of hypothermia and non-invasive ventilation strategies from case-series studies [60]. In this review, no papers were found from the five HICs that provided evidence on the clinical treatment, or the effect of treatment, provided in-hospital to females after drowning. The lack of studies found in the search results may be an indication of the paucity of literature on drowning treatment, especially within emergency medicine.

Outside of the study locations included in this review, two papers [49, 50] were found that reported on the implementation of extracorporeal life support (ECLS) in the intensive care unit after drowning. One study from France reported on patients treated with ECLS after cardiac arrest from drowning. There were only two survivors who survived to six-months after the incident and both were female [49]. The second study [50] included global data from the Extracorporeal Life Support Organization, which collates data on ECLS provided to patients’ in-hospital from over 400 intensive care units worldwide. This study found no significant differences between sexes in outcomes of ECLS post-drowning [50]. In both studies females accounted for 19% [49] and 23% [50] of the study populations, although the sample sizes were both small with less than 50 patients, and both authors declared there were limitations in lack of available data in each study and therefore the interpretation of results may be limited. There is an urgent need for more studies to focus on the clinical treatment provided to patients after drowning, as the number of ED presentations and hospital admissions from a drowning incident among females (and males) are increasing [42].

There is inconsistent and limited information about female’s outcome from care. Within the five HICs included in this review only a small amount of evidence was found from the United States [4, 36–39] on the clinical outcomes of females admitted to hospital after drowning, and patient outcomes were inconsistent. Two studies found females were more likely to experience improved mortality and morbidity [4, 36] and neurological outcome [37] after drowning compared to males. While two other studies found no significant difference between the sexes in survival after drowning [38, 39]. Further evidence on the clinical outcomes of females were found from papers outside the study locations of focus in this review including France, Israel, Korea and Sweden. This included papers on the outcomes of females from cardiac arrest from drowning [20, 51, 52], pneumonia [53, 54], and AKI [21], although the results for the studies are also inconsistent and limited in their wider interpretation.

Female sex was found to be an independent predictor of survival to hospital admission from one study from Sweden [20] which reviewed 529 patients who experienced a cardiac arrest from drowning [20]. Other studies [51, 52, 61] found no difference in the outcomes between females and males in cardiac arrest from drowning. In Claesson et al. [20] females made up 55% of the study population, compared to a smaller proportion of females in the study populations of the other studies 27% [61], 35% [51] and 44% [52].

There is some evidence outside of the study locations on female outcomes of pneumonia [53, 54] and AKI [21] as a result of drowning, with females having better [21, 54] and similar outcomes to males [53]. Although the sample sizes of females in the studies were small, 35% [53], 41% [21] and 46% [54]. In the study from France, seven females were reported to have bacterial pneumonia after drowning in seawater, indicating a more severe respiratory infection, although there was no overall difference in outcomes between females and males [53]. A limitation of this paper is there was no discussion of resuscitation including oxygenation or ventilation support provided to female patients in the intensive care unit with bacterial pneumonia after drowning [53]. A study from Israel on AKI after drowning found females were less likely to experience AKI after drowning compared to males [21]. Females were found to experience a smaller change (from baseline) in their creatinine level within 72 h of drowning compared to males, which may be protective to developing AKI [21]. Although overall there were no significant differences in the outcomes between females and males in this study [21]. Again a limitation of this study on AKI and drowning was a lack of data on the treatment especially resuscitation of patients included in the study [21].

Females who have received lifesaving treatment for the sequelae from drowning have made up a substantial proportion of the study populations in the papers in this review (15 to 55%) [4, 20, 21, 36–39, 49–51, 53, 54, 61]. Despite the variation in sample sizes, they have so far received little attention in drowning literature. The lack of representation of females in clinical studies has been highlighted as an inequity by emergency medicine and gender and sex researchers in recent years [23, 24]. There is a significant gap in knowledge in health research in general on how injury and disease, as well as clinical management including treatment and outcomes, affect females [23, 24]. Some researchers believe this may disadvantage females as there may be crucial differences between females and males that may affect not only the clinical management of the patient but their health outcomes also [23, 24, 62]. In the area of cardiovascular disease research, gender and sex based differences in the clinical management of patients have been thoroughly researched, and critical differences between females and males in the clinical management of cardiovascular disease have been found from pre-hospital care, emergency care, treatment interventions and outcomes [63], however it is unclear how this translates to drowning incidents. Future clinical studies on drowning need to disaggregate all data by gender and sex, specifically in prehospital care, resuscitation and management in ED, and inpatient hospital admission. It is clear more work is needed to ensure treatment options for drowning are optimized for females.

The outcomes from the studies in this review have shown there are results specific to females on the risk factors and the clinical outcomes for drowning, with some studies finding females had better outcomes than males, although overall the results were inconsistent. This review has found unintentional female drowning risk factors and the clinical treatment and outcomes of females admitted to hospital after drowning are inequitably researched and require urgent further exploration. Most studies in this review only reported the clinical outcomes among patients treated for drowning and did not provide evidence or discussion on the clinical treatment given to patients’ in-hospital after drowning. This requires urgent attention within the drowning, emergency and critical care research communities. Patient-centred care has become a crucial component in health care delivery and also health research, advocating for individualised patient care, for this to occur the inclusion of gender and sex based knowledge of injury and disease, and clinical management of such, must be addressed within all areas of health research [24, 64, 65].

### Limitations

The publications included in this review are limited to the search results found from the academic databases accessed during the search strategy. As such, it is possible that some papers may have been missed. Widening the inclusion criteria to other study locations could add to depth of understanding on female drowning, as several papers were found outside the study locations, such as from France, Sweden, Korea and Israel. Excluding grey literature, papers published prior to 2000 and papers not published in English language may have left out other relevant studies on female drowning.

## Conclusion

Adult females are a vulnerable group, which within drowning research has received little attention to date. Despite the overall drowning rate decreasing within the five HICs in focus in this review, the proportion of females who experience unintentional non-fatal and fatal drowning incidents is increasing. There was no single study found that focused on females drowning despite representing one third of drowning incidents world-wide. There is a total lack of evidence on the clinical treatment provided to females in ED and hospital after drowning, and a review of the clinical outcomes from hospitalisation found inconsistent results for females. This review has found female drowning is researched inequitably and thus, there is an urgent need for further exploration of females and unintentional drowning. This includes a need for further studies on risk factors unique to females, and clinical treatment and outcomes of patients treated in hospital for drowning. Drowning prevention strategies must be developed to target females. Such strategies will save the lives of females, a previously neglected group in drowning, while also contributing to an overall reduction in drowning.

## Supplementary Information

**Additional file 1.** Keyword list for search strategy. Keywords used for search strategy.

**Additional file 2.** Supplementary file 1 GRADE evidence profile. GRADE evaluation profile for the included studies.

## Data Availability

Data sharing is not applicable to this study as no datasets were generated or analysed during the current study.

## Abbreviations

AKI: Acute Kidney Injury
ECLS: Extracorporeal Life Support
ED: Emergency Department
GRADE: Grading of Recommendations Assessment, Development and Evaluation
HICs: High Income Countries
ILCOR: International Liaison Committee on Resuscitation
JBI: Johanna Briggs Institute
LMICs: Low- and Middle-Income Countries
NEDS: Nationwide Emergency Department Sample (USA)
NHMRC: National Health and Medical Research Council
PRISMA ScR: Preferred Reporting Items for Systematic Reviews and Meta-Analysis extension for Scoping Reviews
