# Supplementary material for: A scoping review of female drowning: an underexplored issue in five high-income countries
Source: BMC Public Health. 2021 Jun 5;21:1072. doi: 10.1186/s12889-021-10920-8 (PMC8178917; doi:10.1186/s12889-021-10920-8)
Supplement: Supplementary file 1 — Additional file 1. Keyword list for search strategy. Keywords used for search strategy. [file 12889_2021_10920_MOESM1_ESM.docx]

Additional File 1. Keyword list for search strategy (Methods, page 8)

Keywords included:

*Drowning*

‘drown*’, ‘drowning’, ‘near drowning’

AND

*Female*

‘female’

AND

*Sex differences*

‘sex difference’, ‘sex distribution’, ‘gender distribution’, ‘sex factors’, ‘sex ratio’

AND

*Treatment and outcomes*

‘emergency medicine’, ‘emergency treatment’, ‘emergency medical services’, ‘emergency care’, ‘emergency nursing’, ‘hospitalisation’, ‘hospital admission’, ‘treatment outcome’, ‘critical care’, and ‘intensive care’

AND

*Location*

‘Australia’, ‘New South Wales’, ‘Queensland’, ‘Victoria’, ‘Tasmania’, ‘South Australia’, ‘Western Australia’, ‘Australian Capital Territory’, ‘Northern Territory’, ‘New Zealand’, ‘Canada’, ‘United States’, United Kingdom’ and ‘Ireland’.
