# Supplementary material for: A scoping review of female drowning: an underexplored issue in five high-income countries
Source: BMC Public Health. 2021 Jun 5;21:1072. doi: 10.1186/s12889-021-10920-8 (PMC8178917; doi:10.1186/s12889-021-10920-8)
Supplement: Supplementary file 2 — Additional file 2. Supplementary file 1 GRADE evidence profile. GRADE evaluation profile for the included studies. [file 12889_2021_10920_MOESM2_ESM.docx]

Additional File 2. Supplementary file 1 GRADE evidence profile (35) (Methods, page 9)

| Study | Method | Study Limitations | Inconsistency | Indirectness | Imprecision | Publication bias | Quality |
| --- | --- | --- | --- | --- | --- | --- | --- |
| Clemens et al. (2016) (48) | Retrospective descriptive analysis | Serious | Not applicable | No serious indirectness | Not applicable | Not detected | Very low |
| El Sibai et al. (2018) (4) | Retrospective cross-sectional study | Serious | Not applicable | No serious indirectness | Not applicable | Not detected | Very low |
| Gulliver & Begg  (2005) (41) | Cross-sectional study | Very serious | Not applicable | No serious indirectness | Not applicable | Not detected | Very low |
| Henderson & Wilson (2006) (42) | Cross-sectional study | Very serious | Not applicable | No serious indirectness | Not applicable | Not detected | Very low |
| Hudson et al. (2006) (43) | Cross-sectional study | Very serious | Not applicable | Serious | Not applicable | Not detected | Very low |
| Lee et al. (2006) (37) | Retrospective case-series | Serious | Not applicable | No serious indirectness | Not applicable | Not detected | Very low |
| Morgan et al. (2009) (44) | Prospective cross-sectional study | Very serious | Not applicable | No serious indirectness | Not applicable | Not detected | Very low |
| Nasrullah & Muazzam (2011) (45) | Cross-sectional study | Serious | Not applicable | No serious indirectness | Not applicable | Not detected | Very low |
| Peden et al. (2016) (14) | Cross-sectional study | Serious | Not applicable | No serious indirectness | Not applicable | Not detected | Very low |
| Peden et al. (2018) (46) | Prospective Cross-sectional study | Serious | Not applicable | No serious indirectness | Not applicable | Not detected | Very low |
| Peden et al. (2018) (47) | Retrospective Cross-sectional study | Serious | Not applicable | No serious indirectness | Not applicable | Not detected | Very low |
| Quan et al. (2014) (38) | Case-control study | Serious | No serious inconsistency | No serious indirectness | Not applicable | Not detected | Very low |
| Reynolds et al. (2017) (39) | Retrospective Cohort study | Serious | Not applicable | Serious | Not applicable | Not detected | Very low |
| Reynolds et al. (2019) (36) | Retrospective Cohort study | Serious | No serious inconsistency | No serious indirectness | Not applicable | Not detected | Very low |

Legend: GRADE-Grading of Recommendations Assessment, Development and Evaluation
